# Construction and validation of a competing risk model for specific survival of patients with second primary malignancies after prostate cancer

**DOI:** 10.1097/MD.0000000000047400

**Published:** 2026-02-06

**Authors:** Er Hao Bao, Yang Yang, Jia Hao Wang, Yuchen Li, Ying Liu, Lin Yang, Peng Hao

**Affiliations:** a Department of Urology, Sichuan Provincial People’s Hospital East Sichuan Hospital & Dazhou First People’s Hospital, Dazhou, Sichuan, China; b Department of Traditional Chinese Medicine, Sichuan Provincial People’s Hospital East Sichuan Hospital & Dazhou First People’s Hospital, Dazhou, Sichuan, China; c Department of Urology, Nanchong Psychosomatic Hospital, Nanchong, Sichuan, China; d Clinical Medical College, North Sichuan Medical College, Nanchong, Sichuan, China; e Department of Urology, Affiliated Hospital of North Sichuan Medical College, Nanchong, Sichuan, China; f Department of Urology, Dazhou Dachuan District People’s Hospital (Dazhou Third People’s Hospital), Dazhou, Sichuan, China.

**Keywords:** competing risk model, prostate cancer, SPM

## Abstract

To identify second primary malignancies (SPMs) death risk factors in prostate cancer (PCa) survivors and high-risk PCa patients for SPMs. With improved PCa survival, there’s a growing need to study second SPMs in PCa survivors. PCa patients from 2004 to 2015 in the surveillance, epidemiology, and end results database were screened for SPM risk. The Fine and Gray competing risk model identified SPM mortality risk factors via univariate and multivariate analyses. A competing risk nomogram predicted 3-, 5-, and 10-year SPM mortality risk, stratifying patients by total scores for risk assessment. Model performance was assessed using the correlation index, receiver operating characteristic curve, calibration curve, and area under the curve. SPM-diagnosed PCa patients (2004–2015) were split into a 7:3 training (n = 31,435) and validation set (n = 13,472). The nomogram included 12 factors: age, chemotherapy, radiation, Gleason score, race, grade, marital status, tumor size, surgical site, surgery/radiation sequence, scope, and stage. C–index values were 0.70 (se: 0.001) and 0.684 (se: 0.002) in training and validation, respectively, indicating high discriminative power. The 3-, 5-, and 10-year area under the curves in training were 0.75 (95% confidence interval (CI): 0.72–0.77), 0.73 (95% CI: 0.72–0.75), and 0.72 (95% CI: 0.7–0.73), and in validation were 0.7 (95% CI: 0.65–0.74), 0.7 (95% CI: 0.67–0.73), and 0.71 (95% CI: 0.69–0.73), respectively, showing good predictive accuracy. The calibration curve confirmed model fit. A competing risk model predicts SPM mortality in PCa survivors, aiding high-risk patient identification and guiding survival-oriented treatment and follow-up strategies.

## 1. Introduction

Prostate cancer (PCa) is the most frequent cancer in men. Based on a 2019 survey from the National Cancer Institute and the American Cancer Society, PCa ranks first among the most common 2030 cancers.^[1]^ With a poor prognosis, PCa is prone to distant metastases, more than 90% of which occur in bone.^[2]^ Due to more accurate cancer detection and more effective therapies, the 5-year relative survival rate of PCa survivors has been increasing (up to 66%).^[3]^ However, the possibility of developing second primary malignancies (SPMs) has been increasing as well.^[4]^ SPMs, first put forward by Warren et al in 1932,^[5]^ refer to the onset of another cancer with a different pathology after the diagnosis of the primary detected tumor, after a period of time (at least 6 months). Relevant data have shown that the onset of SPMs is not uncommon. About 7% to 15% of cancer survivors may develop SPMs, and the risk of SPMs in cancer survivors is higher (over 20% or so) than that in healthy people.^[6]^ SPMs play a vital role in multiple types of cancer, such as breast cancer,^[7]^ uveal melanoma,^[8]^ and Hodgkin’s lymphoma.^[9]^ SPMs were found to indirectly affect the treatment of PCa.^[10]^

SPMs have become a threat to the lives of PCa survivors. In this context, research on this problem is extremely urgent. However, all-cause death is mostly investigated in the existing studies on risk factors for the death of PCa patients. Long-term follow-ups of PCa patients with a good early prognosis have revealed an increased risk of non-cancer events that may impact the survival and prognosis of those with SPMs after PCa. As current risk assessment tools provide no variables that can reflect other causes of death, the accuracy of prediction tools for all-cause death may be reduced. Actually, specific mortality of other causes is more instructive for clinicians and caregivers than all-cause death. At present, there is a lack of relevant tools to predict the specific mortalities of patients with SPMs after PCa. In this study, the clinicopathological features of patients with SPMs after PCa were comprehensively investigated, and the risk factors related to the progression of SPMs were discussed. In addition, a competing risk model was established to predict the 3-, 5-, and 10-year specific survival of patients with SPMs after PCa.

## 2. Methods

### 2.1. Data collection

Data on patients diagnosed with SPMs after PCa (first primary malignancy: PCa) from 2004 to 2015 were collected from 18 population-based registry studies (2000–2018) in the surveillance, epidemiology, and end results (SEER) database, as data related to prostate-specific antigen and Gleason scores in the SEER database have only been recorded since 2004. These patients were screened based on the 2 key variables (“total number of de novo/malignant tumors” and “sequence number”) in the SEER database. The study population must meet the following inclusion criteria: the first primary malignancy was PCa; SPMs were diagnosed after PCa between 2004 and 2015. The exclusion criteria are: The patients had a medical history of comorbidity of 3 or more malignancies; the patients were diagnosed only by autopsy or death certificate; patients with a life expectancy of <1 month; patients without data related to their age, race, and tumor differentiation degree. Cancer-specific survival (CSS) was the primary endpoint event. Demographic and clinicopathological characteristics of the included patients encompassed race recode (White, Black, and Other), ICD-O-3 Hist/behav (Adenocarcinoma and other), marital status at diagnosis (married and unmarried), Chemotherapy recode (with and without chemotherapy), Radiation recode (with and without radiotherapy), Gleason Score Clinical Recode (≤6, ≥7, and unknown), Grade (high differentiation group and low differentiation group), CS tumor size (≤50, 50–100, >100, and unknown), RX Summ-Surg Prim Site (none and primary site surgery), RX Summ-Surg/Rad Seq (no cancer-directed surgery and/or radiation, radiation before and after surgery, radiation after surgery, and radiation prior to surgery), RX Summ-Scope Reg LN Sur (1–3, ≥4, none), RX Summ-Surg Oth Reg/Dis (none and non-primary surgical procedure), and COMBINED SUMMARY STAGE (localized and regional). This study followed the *Declaration of Helsinki*. As the original data were derived from public databases, medical ethical review was not required.

### 2.2. Data cleaning

Among 588,976 original samples, 45,529 cases were finally eligible for analysis. The following patients were excluded: The primary signal as initial sample (n = 517,889); without data related to marital status, race, risk stratification, surgery information about primary focus, surgery in other regions or at the site of distant metastasis, tumor size, and death status (n = 10,698) at the diagnosis; the subjects with the following surgery information about regional lymph nodes (n = 879); sentinel lymph node biopsy and lymph node resection were provided concurrently; SLNB and lymph node excision were performed at different times; The time of surgery was unknown); the Gleason clinical or pathological scores were not assessed or assessed samples were unknown (n = 13,824); patients whose lymph nodes could not be confirmed positive or negative after detection (n = 134); and patients with a life expectancy of <1 month (n = 15). The remaining 45,529 samples were used for the subsequent analysis.

### 2.3. Competing risk model

All PCa samples were randomized at a 7:3 ratio into the training set (n = 31,435) and validation set (n = 13,472). A chi-square test was performed to validate potential differences in the categorical variables between the training and validation sets. In our competing risk analysis, the cumulative incidence functions (CIFs) of specific mortality and other causes of death were assessed. Univariate and multivariate analyses were conducted to screen out risk factors for the specific mortality of patients with SPMs after PCa. Based on the Fine and Gray models, a competing risk nomogram was established to predict the 3-, 5-, and 10-year specific mortality risks of patients with SPMs. In addition, risk stratification of the entire population was also conducted based on the model. The estimated risk was conducive to identifying high-risk individuals requiring preventive measures. The nomogram’s accuracy and discriminative power were validated using the correlation index (C-index), calibration curve, and area under the curve (AUC).

All data manipulations in this study were carried out in the R software (Version *R*-4.2.1). In terms of all the statistical calculations, a 2-sided *P*-value < .05 was deemed to be statistically significant.

## 3. Results

### 3.1. Basic clinical characteristics

The patients’ baseline characteristics are shown in Table 1. A total of 44,907 PCa patients diagnosed with prostate-specific antigen from 2000 to 2014 were identified from the SEER database, with 21,209 patients (47.2%) in the high tumor differentiation group and 23,689 patients (52.8%) in the low tumor differentiation group. The whites accounted for the largest proportion (83.3%). The tumor sizes of 6296 patients (14%) were <50 mm intraoperatively, and 24,617 patients (54.8%) did not receive primary site surgery. The patients with 1 to 3 lymph nodes resected intraoperatively represented the largest proportion (75.5%); 44,671 patients (99.5%) did not receive chemotherapy; 24,617 patients (54.8%) did not develop lymph node metastasis; it was not the first surgery for only 170 patients (0.4%) to resect distant lymph nodes or other tissues or organs outside the primary site. There were 33,08 patients (7.3%) with a Grade score of ≥7. Significant differences were observed between 18,764 (41.8%) patients who received radiotherapy and 26,143 (58.2%) patients who did not receive radiotherapy (*P* = .008).

**Table 1:** Baseline characteristics.

Factors	Define	Train (N=)	Test (N = 5160)	All (N = 17,200)	*t*/*z (P*)
Marriage	Married	24,647 (78.4)	10,542 (78.3)	35,189 (78.4)	0.134 (.717)
	Unmarried	6788 (21.6)	2930 (21.7)	9718 (21.6)	
Race	White	26,222 (83.4)	11,192 (83.1)	37,414 (83.3)	0.951 (.622)
	Black	3873 (12.3)	1704 (12.6)	5577 (12.4)	
	Other	1340 (4.3)	576 (4.3)	1916 (4.3)	
Grade	High-level	14,811 (47.1)	6398 (47.5)	21,209 (47.2)	0.532 (.470)
	Low-level	16,624 (52.9)	7074 (52.5)	23,698 (52.8)	
ICD	Adenocarcinoma	30,899 (98.3)	13,277 (98.6)	44,176 (98.4)	3.910 (.052)
	Other	536 (1.7)	195 (1.4)	731 (1.6)	
Combine_stage	Localized	26,753 (85.1)	11,403 (84.6)	38,156 (85.0)	1.586 (.210)
	Regional	4682 (14.9)	2069 (15.4)	6751 (15.0)	
Rx_SPS	0	17,264 (54.9)	7353 (54.6)	24,617 (54.8)	0.440 (.508)
	10–90	14,171 (45.1)	6119 (45.4)	20,290 (45.2)	
Rx_SRLS	1 to 3	23,793 (75.7)	10,123 (75.1)	33,916 (75.5)	2.125 (.346)
	≥4	2808 (8.9)	1205 (8.9)	4013 (8.9)	
	None	4834 (15.4)	2144 (15.9)	6978 (15.5)	
Rx_SOR	None	31,323 (99.6)	13,414 (99.6)	44,737 (99.6)	1.378 (.241)
	Non_first_surgery	112 (0.4)	58 (0.4)	170 (0.4)	
Rx_SS	No_radiation	30,286 (96.3)	12,992 (96.4)	43,278 (96.4)	0.500 (.919)
	After	1105 (3.5)	460 (3.4)	1565 (3.5)	
	Before and after	39 (0.1)	17 (0.1)	56 (0.1)	
	Prior	5 (0.0)	3 (0.0)	8 (0.0)	
Radiation	None	18,296 (58.2)	7847 (58.2)	26,143 (58.2)	0.008 (.933)
	Received_radiotherapy	13,139 (41.8)	5625 (41.8)	18,764 (41.8)	
Chemotherapy	None	31,278 (99.5)	13,393 (99.4)	44,671 (99.5)	1.364 (.254)
	Yes	157 (0.5)	79 (0.6)	236 (0.5)	
Gleason	≤6	1236 (3.9)	561 (4.2)	1797 (4.0)	4.960 (.084)
	≥7	2269 (7.2)	1039 (7.7)	3308 (7.4)	
	Unknown	27,930 (88.9)	11,872 (88.1)	39,802 (88.6)	
Tumor_size	≤5o	4365 (13.9)	1931 (14.3)	6296 (14.0)	1.919 (.589)
	50–100	94 (0.3)	36 (0.3)	130 (0.3)	
	>100	26 (0.1)	12 (0.1)	38 (0.1)	
	unknown	26,950 (85.7)	11,493 (85.3)	38,443 (85.6)	

PCa samples (n = 44,907) were randomized into the training set (n = 31,435) and validation set (n = 13,472) at a 7:3 ratio. The training set was used to determine prognostic factors and construct a risk model accordingly. The model was validated in the training and validation sets, respectively. The mean age of patients diagnosed with SPMs was 67.18 ± 8.205 years old in the training set and 67.15 ± 8.277 years old in the validation set, respectively. There was no statistical difference in baseline characteristics between the 2 sets, as shown in Table 1.

### 3.2. Influencing factors related to poor outcomes of patients with SPMs after PCa

**Figure 1.**
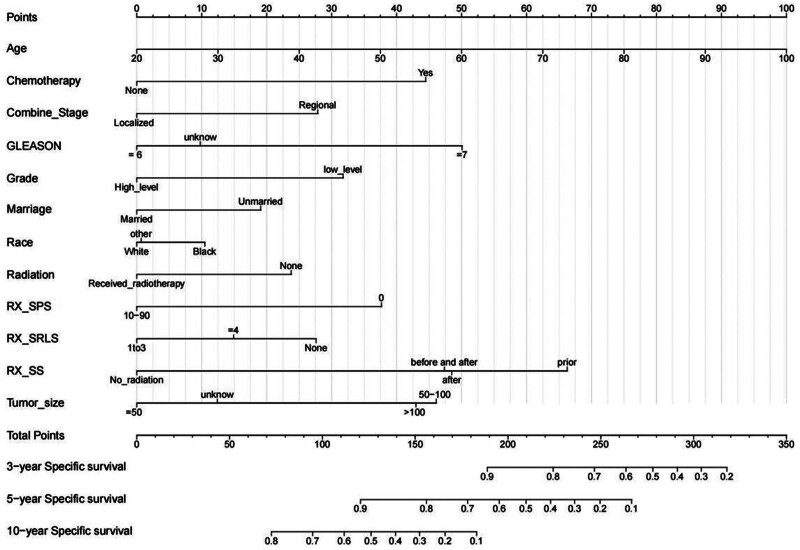
Column diagram. RX_SPS: RX Summ – Surg Prim Site (None and Primary site surgery) RX_SRLS: RX Summ – Surg/Rad Seq (No cancer-directed surgery and/or radiation, Radiation before and after surgery, Radiation after surgery, and Radiation prior to surgery) RX_SS: RX Summ – Scope Reg LN Sur (1–3, ≥4, None)

The results of the univariate regression conducted in the training set revealed that age recode with single ages and 100, Race recode, ICD-O-3 Hist/behav, Chemotherapy recode, Radiation recode, Gleason Score Clinical Recode, Grade, CS tumor size, RX Summ-Surg Prim Site, RX Summ-Surg/Rad Seq, RX Summ-Scope Reg LN Sur, and RX Summ-Surg Oth Reg/Dis were associated with the poor prognosis of patients with SPMs after PCa. The CIF curve presented that the cumulative rate of specific mortality in PCa patients rose with the increase in the follow-up time (Fig. 1).

The multivariate analysis results revealed that survival-related prognostic variables included age (HR = 1.02, 95% confidence interval [CI] = 1.01–1.03), race (with the whites as a reference, the Blacks: HR = 1.16, 95% CI = 1.03–1.32; Others: HR = 1.03, 95% CI = 0.84–1.26), marital status (with the married as a reference, the unmarried: HR = 1.4, 95% CI = 1.27–1.55), chemotherapy (with the patients who did not receive chemotherapy as a reference, those who received chemotherapy: HR = 2.51, 95% CI = 1.74–3.63), radiotherapy (with the patients who did not receive radiotherapy as a reference, those who received radiotherapy: HR = 0.69, 95% CI = 0.62–0.77), Gleason score (with the patients with the Gleason score of ≤6 as a reference, those with the Gleason score of ≥7: HR = 3.05, 95% CI = 1.67–5.58), tumor differentiation degree (with the patients with high tumor differentiation degree as a reference, those with low tumor differentiation degree: HR = 1.81, 95% CI = 1.65–2), primary site surgery (with the patients who did not receive PCa-related surgery as a reference, those who received PCa-related surgery: HR = 0.48, 95% CI = 0.41–0.56), number of intraoperatively resected lymph nodes (with the patients in whom the number of intraoperatively resected lymph nodes was 1 to 3 as a reference, those in whom the number was ≥4: HR = 1.35, 95% CI = 1.07–1.7), sequence of surgery and radiotherapy (with the patients who did not receive radiotherapy as a reference, those who received postoperative radiotherapy: HR = 2.69, 95% CI = 2.15–3.36; patients who received preoperative and postoperative radiotherapy: HR = 3.93, 95% CI = 1.24–1.26; those who received preoperative radiotherapy: HR = 3.3, 95% CI = 1.34–8.16), and tumor size (with the subjects having a tumor size of ≤50 mm as a reference, those with a tumor size of 50 to 100 mm: HR = 2.02, 95% CI = 1.02–4.01; a tumor size of >100 mm: HR = 1.92, 95% CI = 0.48–7.74). These clinical prognostic variables were all incorporated into a competing risk nomogram model for further analysis (Table 2).

**Table 2:** Univariate and multivariate analyses.

		Univariate		Multivariate	
Factors	Define	HR (95% CD)	Z (*P*)	HR (95% CI)	*Z (P*)
Age	Age	1.03 (1.03–1.04)	0	1.02 (1.01–1.03)	
Chemotherapy	Yes	3.32 (2.32–4.75)	0	2.51 (1.74–3.63)	
	No				
Combine_Stage	Localized				
	Regional	1.38 (1.23–1.54)	0	1.73 (1.51–1.98)	
Gleason	<6				
	≥7	4.51 (2.47–8.23)	0	3.05 (1.67–5.58)	3.00E−04
	Unknown	4.68 (2.65–8.25)	0	2.72 (1.53–4.83)	0.0006
Grade	High-level				
	Low-level	1.87 (1.71–2.05)		1.81 (1.65–2)	
ICD	Adenocarcinoma				
	Other	1.17 (0.87–1.57)	0.31	NA	NA
Marriage	Married				
	Unmarried	1.53 (1.4–1.69)	0	1.4 (1.27–1.55)	
Race	White				
	Black	1.25 (1.11–1.42)	3.00E−04	1.16 (1.02–1.32)	0.02
	Other	1.14 (0.93–1.4)	0.2	1.03 (0.84–1.26)	0.79
Radiation	None				
	Received_radio	1.19 (1.09–1.3)	1.00E−04	0.69 (0.62–0.77)	
Rx_SOR	None				
	Non_first_surgery	1.51 (0.81–2.83)	0.2	NA	NA
Rx_SPS	0				
	10–90	0.59 (0.54–0.65)	0	0.48 (0.41–0.56)	
RX_SRLS	1to 3				
	≥4	1.45 (1.15–1.84)	0.0017	1.35 (1.07–1.7)	0.013
	None	1.97 (1.61–2.41)	0	1.67 (1.33–2.08)	0
Rx_SS	No_radiation				
	After	1.79 (1.49–2.15)	0	2.69 (2.15–3.36)	0
	Before and after	3.02 (0.5–18.27)	0.23	3.93 (1.24–12.461	0.02
	Prior	1.95 (0.79–4.79)	0.15	3.3 (1.34–8.16)	0.0097
Tumor_size	≤50				
	50–100	2.92 (1.52–5.59)	0.0013	2.02 (1.02–4.01)	0.045
	>100	1.83 (0.44–7.58)	0.4	1.92 (0.48–7.74)	0.36
	Unknown	1.6 (1.37–1.87)	0	1.24 (1.05–1.46)	0.011

RX_SPS: RX Summ – Surg Prim Site (None and Primary site surgery).

RX_SRLS: RX Summ – Surg/Rad Seq (No cancer-directed surgery and/or radiation, Radiation before and after surgery, Radiation after surgery, and Radiation prior to surgery).

RX_SS: RX Summ – Scope Reg LN Sur (1–3, ≥4, None).

RX_SOR: RX Summ – Surg Oth Reg/Dis (None and Non-primary surgical procedure).

### 3.3. Construction and validation of the competing risk nomogram for specific survival of patients with SPMs after PCa

**Figure 2.**
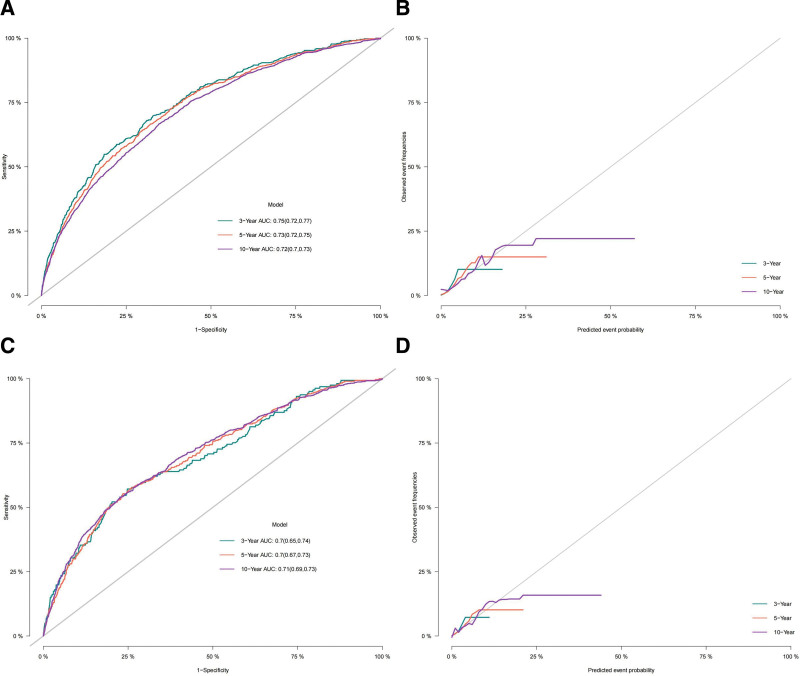
(A) ROC curve and (B) calibration curve of training set, (C) ROC curve and (D) accuracy curve of verification set. AUC = area under the curve, ROC = receiver operating characteristic.

Based on the univariate and multivariate analyses, the predictive factors with *P* < .05 were selected to establish a competing risk nomogram model to predict the 3-, 5-, and 10-year specific survival of patients with SPMs after PCa. The following 12 clinical indicators were included in our competing risk model: age recode with single ages and 100, Chemotherapy recode, Radiation recode, Gleason Score Clinical Recode, Race recode, Grade, Marital status at diagnosis, CS tumor size, RX Summ-Surg Prim Site, RX Summ-Surg/Rad Seq, RX Summ-Scope Reg LN Sur, and COMBINED SUMMARY STAGE. In order to assess the discriminative capability of the constructed competing risk model, its accuracy was assessed using the C-index. The C-index values of the nomogram in the training and validation set were 0.70 (se: 0.001) and 0.684 (se: 0.002), respectively, indicating that the model had high discriminative power. ROC curves were plotted to analyze the AUCs in the training and validation sets (as shown in the figures below). The AUCs for predicting the 3-year, 5-year, and 10-year CSS were 0.75 (95% CI: 0.72–0.77), 0.73 (95% CI: 0.72–0.75), and 0.72 (95% CI: 0.7–0.73) in the training set, and 0.7 (95% CI: 0.65–0.74), 0.7 (95% CI: 0.67–0.73), and 0.71 (95% CI: 0.69–0.73) in the validation set, respectively. The model’s prediction fits the actual observation as well as the calibration curve indicates (Fig. 2).

The model’s prediction fits the actual observation as well as the calibration curve indicates (Figure 2).

### 3.4. Risk stratification

The individual score of each patient was calculated according to the established competing risk model, and the optimal critical value was obtained based on the median score. As shown in the CIF curve (Figure S1, Supplemental Digital Content, https://links.lww.com/MD/R264), the 3-, 5-, and 10-year CSS of the low-risk prognosis group is significantly higher than that of the high-risk group, and the survival prognosis of those in the high-risk group was very poor.

### 3.5. Comparison of mortality estimation between traditional survival analysis and competing risk model

As shown in the figure below, the 3-, 5-, and 10-year mortality risks estimated through traditional survival analysis were 2.37%, 4.41%, and 10.43% respectively, while the cumulative specific mortality risks predicted by the competing model were 1.34%, 2.49%, and 5.75% respectively. The results showed a big difference in the mortalities estimated by traditional survival analysis and the competing risk model. In this study, the mortality estimated through the conventional method was higher than that predicted by the competing model. Since the PCa patients who died from other causes accounted for a relatively large proportion in this study, the specific mortality of PCa patients may be overestimated in traditional COX regression analysis (Table S1, Supplemental Digital Content, https://links.lww.com/MD/R264).

## 4. Discussion

A total of 45,529 patients with SPMs after PCa were included in this study. Based on univariate and multivariate analyses, the risk factors for specific death included age recode with single ages and 100, Chemotherapy recode, Radiation recode, Gleason Score Clinical Recode, Race recode, Grade, Marital status at diagnosis, CS tumor size, RX Summ-Surg Prim Site, RX Summ-Surg/Rad Seq, RX Summ-Scope Reg LN Sur, and COMBINED SUMMARY STAGE. A competing risk model was established to forecast the 3-, 5-, and 10-year specific mortality risks of patients with SPMs after PCa. It can be used to guide the determination of symptomatic treatment and rehabilitation regimens and predict risks, so that government departments and health administrators can better manage medical quality and rationally allocate medical resources. The model was tested using the C-index and calibration curve, which showed good accuracy and calibration.

Several independent variables that may affect the outcome of patients with SPMs were identified by the competing risk model, such as radiotherapy. Nevertheless, its impact on PCa survivors with SPMs is still controversial. Earlier studies have revealed that PCa patients receiving radiotherapy may be more susceptible to developing SPMs.^[11]^ A study analyzed data on many PCa patients from public databases to compare the risk of SPMs between the radiotherapy and the non-radiotherapy groups, and the results indicated that radiotherapy may indeed increase the risk of some types of SPMs. In this regard, strengthening monitoring should be considered.^[12]^ Meanwhile, other studies have reported no difference in the incidence of SPMs between patients receiving radiotherapy and other treatments.^[13]^ Zhumei Zhan et al believed that chemotherapy drugs could also further elevate the risk of SPMs in patients with non-Hodgkin’s lymphoma.^[14]^ The correlation between marital status and patient survival of multiple cancers was explored, including non-small cell lung cancer^[15]^ and colorectal cancer.^[16]^ A summary analysis of 12 case-controlled studies from the practical impact alliance has revealed that single men have a higher risk of high-grade cancer than married men or men who have female companions.^[17]^ Disease staging, tumor grading, and the quantity of lymph nodes involved are prognostic factors for PCa patients.^[18]^ However, the degree of pelvic lymphadenectomy during radical prostatectomy still remains a controversial topic.^[19]^

A competing risk model established through regression analysis has been widely applied to predict the prognosis of various cancers.^[20]^ A study constructed a model based on the following 7 independent factors to predict the 3- and 5-year overall survival of SPM patients: sites of SPMs, age, tumor node metastasis classification, surgery history of SPMs, and PCa staging.^[10]^ Nonetheless, most of the deaths were found to be induced by SPMs rather than PCa.^[21]^ Accordingly, there would be a competing risk bias. Based on the comparison of mortality estimation between traditional survival analysis and competing risk model, the risk of mortality may be overestimated by traditional survival analysis methods (K-M method and Cox proportional risk regression model). The competing risk model constructed in this study applies to multiple endpoints, which can process survival data on multiple potential outcomes and avoid competing risk bias, thus improving the accuracy of the risk model and achieving more accurate predictions.

This study has the following limitations. Firstly, this study is retrospective, and thus our findings need to be further demonstrated by future prospective studies. Secondly, detailed treatment information was not available in the SEER database, such as the exact sites and surgery history of SPMs. Thirdly, most of the participants in this study were whites. Hence, more multicenter clinical pilot studies are warranted to determine whether the study results can be generalized to different groups.

## 5. Conclusion

Age recode with single ages and 100, Chemotherapy recode, Radiation recode, Gleason Score Clinical Recode, Race recode, Grade, Marital status at diagnosis, CS tumor size, RX Summ-Surg Prim Site, RX Summ-Surg/Rad Seq, RX Summ-Scope Reg LN Sur, and COMBINED SUMMARY STAGE are determined as independent predictive factors for CSS of patients with SPMs after PCa. The constructed model has good accuracy and high identification power, which can accurately predict the risk of mortality and help doctors and patients make clinical decisions. Moreover, it can also provide a reference basis for the clinical classification and stratification of diseases, which is conducive to formulating prognostic treatment and follow-up strategies beneficial for the survival of patients at different risk levels.

## Author contributions

**Conceptualization:** Jia Hao Wang.

**Formal analysis:** Er Hao Bao, Yang Yang.

**Funding acquisition:** Ying Liu.

**Investigation:** Er Hao Bao, Yang Yang.

**Methodology:** Er Hao Bao,Yang Yang.

**Writing–review & editing:** Peng Hao.

**Writing - original draft preparation:** Er Hao Bao, Lin Yang.

## Supplementary Material


