# Supplementary material for: Construction and validation of a competing risk model for specific survival of patients with second primary malignancies after prostate cancer
Source: Medicine (Baltimore). 2026 Feb 6;105(6):e47400. doi: 10.1097/MD.0000000000047400 (PMC12885679; doi:10.1097/MD.0000000000047400)

Supplementary Table:

Supplementary Table 1 Comparison of competitive risk models

| Time | **Traditional Survival Analysis** | **Competing Risk Model** | |
| --- | --- | --- | --- |
|  |  | Specific Mortality | Mortality from Other Causes |
| 12 | 0.57 | 0.33 | 1.34 |
| 24 | 1.38 | 0.78 | 3.94 |
| 36 | 2.37 | 1.34 | 7.23 |
| 48 | 3.36 | 1.9 | 10.79 |
| 60 | 4.41 | 2.49 | 14.83 |
| 72 | 5.55 | 3.12 | 18.69 |
| 84 | 6.69 | 3.75 | 23.12 |
| 96 | 7.86 | 4.39 | 27.49 |
| 108 | 9.18 | 5.1 | 32.05 |
| 120 | 10.43 | 5.75 | 36.85 |
| 132 | 11.69 | 6.39 | 41.36 |
| 144 | 13.04 | 7.04 | 45.82 |
| 156 | 14.3 | 7.62 | 49.98 |
| 168 | 15.29 | 8.05 | 54.04 |
| 178 | 16.23 | 8.45 | 56.88 |

**Supplementary Figure:**

Supplementary Figure 1 Nelson-Aalen cumulative hazard curve of the competing risk model


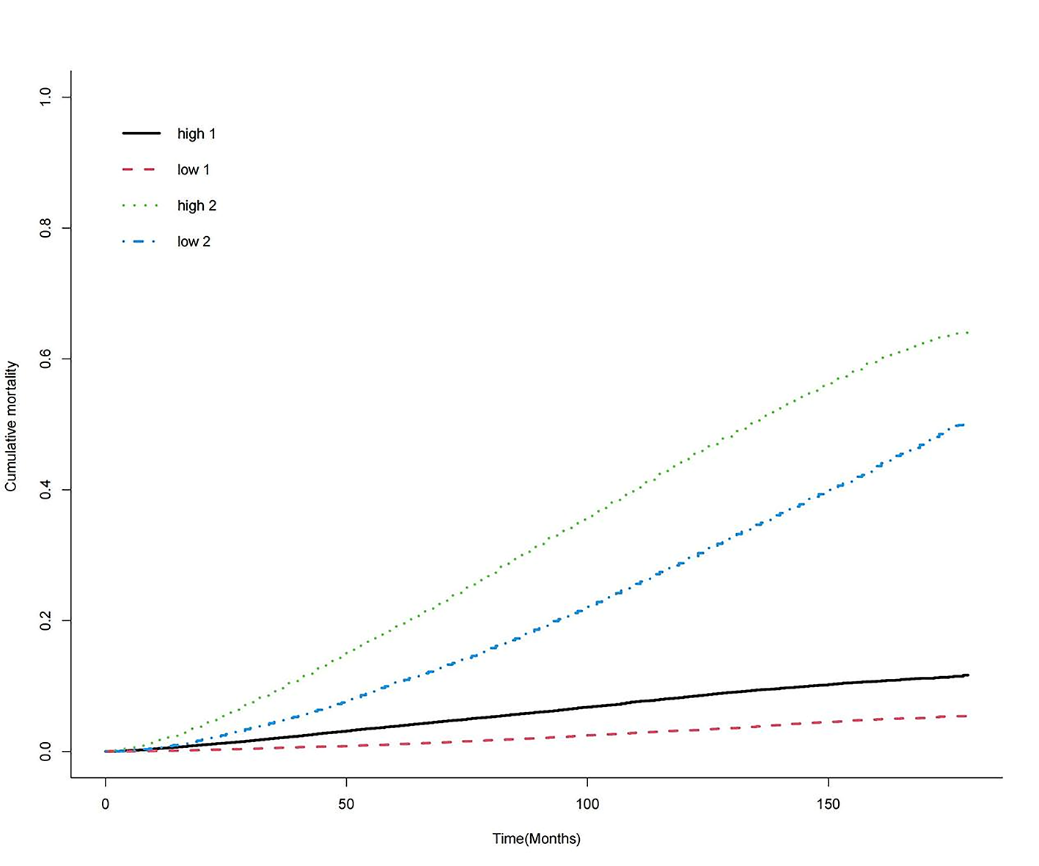

Supplement: Supplementary file 1 [file medi-105-e47400-s001.docx]
